# Matching STR and SNP genotyping to discriminate between wild boar, domestic pigs and their recent hybrids for forensic purposes

**DOI:** 10.1038/s41598-020-59644-6

**Published:** 2020-02-21

**Authors:** Rita Lorenzini, Rita Fanelli, Francesco Tancredi, Antonino Siclari, Luisa Garofalo

**Affiliations:** 1 Istituto Zooprofilattico Sperimentale delle Regioni Lazio e Toscana “M. Aleandri”, Centro di Referenza Nazionale per la Medicina Forense Veterinaria, Via Tancia 21, 02100 Rieti, Italy; 2 Istituto Zooprofilattico Sperimentale delle Regioni Lazio e Toscana “M. Aleandri”, Via Tancia 21, 02100 Rieti, Italy; 3 Ente Parco Nazionale dell’Aspromonte, Via Aurora 1, 89057 Gambarie di S. Stefano in Aspromonte, Reggio Calabria, Italy

**Keywords:** PCR-based techniques, Genetic markers

## Abstract

The genetic discrimination between phylogenetically close taxa can be challenging if their gene pools are not differentiated and there are many shared polymorphisms. The gene flow between wild boar (*Sus scrofa*) and domestic pig (*S. s. domesticus*) has never been interrupted from domestication onwards, due to non-stop natural and human-mediated crossbreeding. To date there are no individual genetic markers that are able to distinguish between the two forms, nor even to identify effectively their hybrids. We developed a combined molecular protocol based on multiplex porcine-specific STR-profiling system and new real time PCR-based assays of single polymorphisms in the *NR6A1* and *MC1R* genes to gain high diagnostic power in the differentiation of wild boar, pig and hybrids for forensic purposes. The combined approach correctly assigned individuals to one or the other parental gene pool and identified admixed genotypes. Evidence was found for substantial reduction of false negative results by using multiple marker systems jointly, compared to their use individually. Our protocol is a powerful and cost-effective diagnostic tool that can easily be adopted by most forensic laboratories to assist authorities contrast food adulteration, assure veterinary public health and fight against wildlife crimes, like poaching and illegal detention of wild animals.

## Introduction

The genetic identification of interspecific hybrids is a powerful tool in zoological and evolutionary scientific research, as well as in conservation and animal forensics^1,2^ [for reviews]. The need for a diagnostic tool to identify hybrids is how much ever pressing in wildlife forensics. For example, the DNA-based recognition of wild and domestic parental forms from the same species, along with their hybrids (e.g crossbreeds of wolf and dog, sheep and mouflon or wild boar and domestic pig), is an effective diagnostic tool to support the authorities in the persecution of poaching and illegal detention of wild animals^3–7^. According to the European Council Regulation (EC) n. 338/97^8^, hybrids up to the fourth generation (i.e. that have in their previous four generations of the lineage one or more parentals of the wild species) are referred to as wild species under the view of CITES (Convention on International Trade in Endangered Species of Wild Fauna and Flora) protection: when comprised in Annexes A or B, they are subject to the precepts of the Regulation just as if they were full species.

The ability to distinguish wild boar (*Sus scrofa*), domestic pig (*S. s. domesticus*) and their hybrids is no doubt important for conservation, but it is also a valuable tool in the field of food frauds^9–11^. The substitution of wild boar meat with lower quality but cheaper meat from domestic pig can be a frequent practice of suppliers and food manufactures that fraudulently mislabel their products. However, the genetic identification of two phylogenetically close taxa such as wild boar and pig is complicated, as the gene flow between the two, due to natural and human-mediated crossbreeding, has never been interrupted since domestication (from 6000 to 10000 years ago^12–14^) to date. For that very reason, their hybrids are often cryptic or hardly disting uishable and the phenotypic makeup is not helpful for settling the question. Furthermore, hybridization can reach high levels in local wild populations, like in southern Italy (e.g. Campania, Calabria) or eastern Europe^14–16^.

The analysis of highly variable autosomal Short Tandem Repeats (STRs) has been widely used to infer genetic variation in wild and domestic populations^2,17^. Currently, STRs are the markers of choice for assessing levels of hybridization between gene pools of different taxa^18–20^. Nevertheless, when taxa are genetically very closely related, sharing several alleles with similar population frequencies and few fixed differences, the diagnostic power of a standard panel of STR loci may be insufficient to minimize type II errors (false negatives) and prevent many hybrids from going undetected.

The melanocortin-1 receptor (*MC1R*) at the Extension (E) autosomal locus plays a key role in the phenotypic variation of the coat/plumage colour in many mammal and bird species^13,21^. Sequencing of *MC1R* on chromosome 6 in *S. scrofa* revealed a series of single-base substitutions corresponding to different E alleles with phenotypic effects^21,22^. The wild type allele E^+^ (or 0101, *sensu*^13^), determining a typical brownish-grey coat colour, is private to the European and Asian wild boar populations, and to the Hungarian Mangalica swine breed^13,14^. The variants E^D2^ (0301), E^P1/2/3^ (0501/0502/0503) and the recessive allele *e* (0401) are of domestic European origin, and determine different coat colours, from black to spotted, white, and reddish. Additionally, the alleles E^D1^ (0201/0202/0203), fixed in Asian pigs, are associated to a typical black coat colour.

Two single-base allelic variants at the nuclear receptor subfamily 6, group A, member 1 gene (*NR6A1*), mapped on chromosome 1, affect the number of vertebrae in *S. scrofa*^23^. Wild boars have typically 19 thoracic and dorsal vertebrae, that grow in number to 21–23 in pig, following the enlargement of body size for meat production and improvement of reproductive performances that accompanied domestication^23^. The *NR6A1* gene sequence contains one diagnostic site corresponding to a missense mutation (C > T) that yields the substitution of a proline in wild boars, with a leucine in the European pigs^23^. The wild C allele is also present at low frequency (0.250) in Iberian pigs, while it reaches frequencies as high as fixation in Asian domestic pigs (0.760–1.00^24,25^). Chinese native breeds show the T allele only rarely^26^. The presence of wild and domestic variants in CT heterozygotes reveals the presence of hybrids and consequently the occurrence of bidirectional introgression, even though it cannot be traced whether heterozygotes represent recent (i.e. F1s) or advanced-generation hybrids (i.e. interbreeds among hybrids or later-generation backcrosses).

To date, variation at *MC1R* and *NR6A1* genes has been used to identify wild boar × pig hybrids only rarely, with the different alleles having been identified through RFLP (Restriction Fragment Length Polymorphism) analysis of partial gene sequences amplified *via* PCR^11,27–29^. The method is efficient in discriminating all alleles, but it is relatively expensive (due to use of different restriction enzymes each revealing different point mutations), as well as highly time-consuming and laborious in the interpretation of data.

Purpose of the present study is to develop a molecular protocol for the discrimination of wild and domestic gene pools of *S. scrofa* and the identification of admixed genotypes by: 1) setting up a multiplex STR-typing test for the assignment of individuals to their parental population through Bayesian clustering algorithm; 2) designing fast and low-cost qualitative real time PCRs using new sets of specific primers/probes to detect base variation (Single Nucleotide Polymorphisms, SNPs) at four diagnostic sites in the *MC1R* and *NR6A1* genes; 3) testing the efficacy of the two marker systems, used separately and as a joint approach, in the identification of hybrids.

We ultimately aim for a combined molecular method that significantly increases the diagnostic power, compared to the two marker systems used individually, and that features a fair compromise between costs and benefits. Forensic applications of our protocol range from contrasting food frauds to fighting against poaching and the illegal detention of wild animals.

## Materials and Methods

### Samples and DNA extraction

To develop our STR- and SNP-based tests we gathered muscle and blood samples of 83 unrelated pigs and 164 wild boars to use as reference populations. Pigs from commercial breeds (Landrace, Large White, and crossbreeds between the two or with Duroc) were collected in small-scale farms as well as in factory farms for the large-scale distribution on markets of northern and central Italy. We have focused our sampling on common commercial pigs, rather than on rustic or valuable breeds, since the latter do not contribute to introgression of domestic genes into the wild populations, as breeders have a high interest in rearing them in purity and keep their animals under strict control. Of 164 wild boars, 137 were collected in central (Latium, Tuscany, Umbria, Marche) and southern Italy (Calabria), and 27, belonging to the subspecies *S. s. meridionalis*, were from Sardinia. All wild boars came from legal hunting and were provided without any detail on their phenotypes. Populations in Sardinia and Calabria are known to be introgressed with domestic pigs^15^. We deliberately sampled wild boars also from those areas, in order to set up and validate our tests on all genotypes, homozigotes and heterozygotes, that would have reasonably been present in the dataset. To validate the tests on “field samples” we also collected 6 wild boar × domestic pig hybrids with known genealogy (F1s, pig and wild boar backcrosses) and 15 putative hybrids (pigs showing wild morphological traits and wild boars showing domestic traits) with no genealogical information. Hybrids with known genealogy were collected during regular disease monitoring in small farms where veterinarians accidentally found that pigs had been illegally interbred with wild boars. Putative hybrids were either seized animals (suspected to be wild boars illegally kept in captivity) or free roaming pigs found dead in the field, in areas (Calabria) where domestic pigs are frequently reared in free or semi-free conditions, and live in sympatry with wild boars. Unusual morphological traits, as clues of hybridization, were evaluated by zoologists in the wild, and by trained veterinarians in farms.

DNA was isolated from approximately 25 mg of muscle or 25 µl whole blood using the QIAamp DNA Mini Kit (Qiagen, Hilden, Germany), following the manufacturer’s instructions. DNA quantification was obtained with the QuantiFlor^®^ dsDNA system (Promega, Madison, USA).

### Molecular procedures and analysis of data

#### STR typing

Twenty unlinked autosomal STRs were amplified in five multiplexes (M1-M5) to determine individual multilocus genotypes. The following STR markers were chosen according to the guidelines of the International Society of Animal Genetics–Food and Agriculture Organization (ISAG–FAO) Advisory Committee for parentage control and linkage mapping in swine: S0155, SW122, SW911, SW936 (M1); S0005, S0026, SW461, SW632, SW951 (M2); SW2496, SW2021, S0068, SW857 (M3); SW2532, SW0097, SW240, SW72 (M4); SW24, S0090, SW1492 (M5). Primer pairs and updated details on STR loci can be found in the database for pigs or at the website www.fao.org/docrep/014/i2413e/i2413e00.pdf. Each multiplex contained 30 ng of total DNA, 0.2 µM of each primer (with the forward primer fluorescently labeled) and 12,5 µl of multiplex mix (Qiagen Multiplex PCR Kit) in 25 µl total volume. PCRs were accomplished on Veriti 96 Well thermal cycler (Applied Biosystems, Foster City, CA, USA), heated to 95 °C for 15 min, subjected to 32 cycles at 94 °C for 30 s, the annealing temperature of 58 °C for 90 s, and to an extension at 72 °C for 60 s. The final synthesis step was extended to 30 min at 60 °C. Seven-fold diluted multiplexes were loaded onto an ABI Prism^TM^ 3130 Genetic Analyzer (Applied Biosystems), and fragment sizing was performed using the GeneScan-500 LIZ size standard. GeneMapper Software 5.0 was used to score the alleles.

We applied the model-based Bayesian clustering algorithm as implemented in the software STRUCTURE version 2.3.4^30^ to assign individuals to their source population, and to identify genotypes with putative admixed ancestry. The method utilizes the information from highly variable STR loci and sorts multilocus genotypes into clusters of genetically similar individuals, inferring K theoretical populations that are in Hardy-Weinberg equilibrium and show linkage equilibrium between loci. Reference wild boars and pigs were initially considered as a single sample, without any *a priori* classification of individuals (i.e. without prior non-genetic information on populations) using the admixture model with default settings and correlated frequencies between populations as priors. Five independent runs with value of K varying from 1 to 10 were performed to test for consistency of results, using 5 × 10^6^ iterations and 5 × 10^5^ steps of burn in to achieve convergence. We explored the most probable value of K with the Δk method developed by Evanno *et al*.^31^ using STRUCTURE HARVESTER^32^, together with other information provided by STRUCTURE, like the posterior probability of the data for a given K itself, L(K), the value of α and the individual assignment patterns (cf.^31,33^). Ten additional runs were carried out based on the derived K value, and assignment results from the run with the highest L(K) value were selected^34^. Eventually, the average proportional membership (Q) of wild boar and pig populations, along with the proportional membership (q_i_) of single multilocus genotypes, were derived for each of the inferred clusters.

We chose a threshold of posterior probability for cluster membership of q > 0.90 to assign individuals as genetically pure pigs or pure wild boars, so that individuals showing q ≤ 0.90 in their respective cluster(s) were identified as hybrids. This cut-off was selected to avoid false positives, i.e. to avoid that pure individuals would be erroneously classified as hybrids, as one might risk using a low cut-off (^6^ and ref. herein), especially when Fst < 0.21^35^ and when parental gene pools share many STR alleles, as in our case. Furthermore, no reference pigs (except two, but see Results) showed q_i_ values greater than 0.10 in the wild boar clusters.

To test for the proportional membership q_i_ of hybrids with known genealogy and putative hybrids in each derived cluster, all samples were re-analyzed with STRUCTURE under same settings as above, this time assuming that pig and wild boars were *a priori* correctly identified and assigned to their own clusters (option *popflag* = *1*). By contrast, known hybrids and putative hybrids were left unassigned (*popflag* = *0*). The latter, together with two pigs and four wild boars that were identified as genetically admixed by previous analyses on reference samples (see Results), were excluded from allele frequency calculations (option *update allele frequencies using only individuals with popflag* = *1*).

#### Set up of SNP assays by real time PCR

We developed duplex real time PCRs using TaqMan probes for allele discrimination at three diagnostic sites in the *MC1R* locus and at one site in the *NR6A1* gene of *S. scrofa* (Table 1). To set up real time PCRs on the diagnostic sites of *MC1R* we aligned 22 wild boar and pig sequences downloaded from GenBank (Table S1), and manually designed three sets of primers/probe. Referring to as the reference *MC1R* pig sequence EU443645, an assay was first designed on position 370 (MC1R_1, G > A), which discriminates allele E^+^ (G), typical of wild boar, from allele E^D2^ (A) and alleles E^P1/2/3^ (A), both present in European pigs, but not from allele E^D1^ (G), which is typical of Asian domestic pigs, and not from the recessive allele *e* (G), which is found in the reddish Duroc pigs. Variation at site 727 (MC1R_2, G > A) distinguishes the allele *e* (A) from all the other alleles E^+^, E^D1^, E^D2^, E^P1/2/3^ (G). Mutation at a third SNP, corresponding to position 729 (MC1R_3, G > A), discriminates allele E^D1^ (A) from alleles *e*, E^+^, E^D2^ and E^P1/2/3^ (G). The combination of genotypes at the three SNPs allows us to identify all the above alleles, with the exception of E^P1/2/3^ from E^D2^. However, for our purposes, it is not crucial that these two alleles be separated. A fourth real time PCR assay was developed based on the *NR6A1* polymorphism C > T at site 748 (reference pig sequence AB248749^23^). The wild-type allele C is typically present in the European wild boars, Iberian and Asian native pigs. A mutation C > T is fixed in European domestic pigs. The guide of the Primer Express software 3.0.1 (Applied Biosystems) was followed to search for best *in silico* primer location, efficiency of binding and melting temperatures of our primers/probe sets. Sequences of the designed primers and probes were checked for specificity of binding in the expected genes of *S. scrofa* through the BLAST algorithm (https://blast.ncbi.nlm.nih.gov/Blast.cgi). PCR reactions contained 5 µl TaqMan GTXpress^TM^ Master mix (Applied Biosystems, Life Technologies, part number 4401890), 900 nM each primer, 200 nM probes (FAM/MGB- and VIC/MGB-labeled TaqMan® Probes by Applied Biosystems, Life Technologies) and 30 ng DNA in 10 µl total volume. Amplification profiles consisted of a hold stage of 20 s at 95 °C, a PCR stage of 40 cycles at 95 °C for 15 s and 60 °C for 1 min, with a final post-read stage of 30 s at 25 °C. Thermal cycling was performed in a QuantStudio 7Flex (Applied Biosystems, Life Technologies). One positive control (heterozygous genotype with target DNA at 30 ng) and one negative control (reagents with no template) were included in each amplification run.

**Table 1:** Primers and probes of real-time-PCR SNP assays at three positions in the *MC1R* gene and one position in *NR6A1* for discrimination of wild and domestic *Sus scrofa*.

SNP name	SNP Position	Primer sequences (5′-3′)	TaqMan probes	Amplicon size (bp)	Alleles
MC1R-1	370	F-GTGAGCGTGAGCAACATGCT R-CACCATGGAGCCGCAGAT	6FAM-ACGTCATG***g***ACGTGC-MGB NFQ VIC-CGTCATG***a***ACGTGC-MGB NFQ	129	**E**^**+**^, E^D1^, *e* E^D2^, E^P1/2/3^
MC1R-2	727	F-CGCCCGGCTCCACAA R-ACGCCCAGCAGGATGGT	6FAM-CTCAAGGGC***g***CGGC-MGB NFQ VIC-CAAGGGC***a***CGGCC-MGB NFQ	84	**E**^**+**^, E^D1^, E^D2^, E^P1/2/3^ *e*
MC1R-3	729	F-CGCCCGGCTCCACAA R-ACGCCCAGCAGGATGGT	6FAM-CTCAAGGGCGC***g***GC-MGB NFQ VIC-CTCAAGGGCGC***a***GC-MGB NFQ	84	**E**^**+**^, E^D2^, E^P1/2/3^, *e* E^D1^
NR6A1	748	F-AGGGCTTCAGAGAGCAACCA R-TGAAGCTCACCTGGAGGACAGT	VIC-TCAC**c**GGGCTCCA-MGB NFQ 6FAM-CTCAC***t***GGGCTCC-MGB NFQ	60	**C** T

Diagnostic sites in TaqMan probes are in italic lowercase and underlined. Corresponding alleles are also indicated. Wild alleles are in bold. SNP positions are according to the reference pig sequences EU443645 (*MC1R*) and AB248749 (*NR6A1*). F = forward primer, R = reverse primer.

Default settings were used to calculate Ct values for alleles (threshold = 0.2, automatic baseline start cycle 5, end cycle 15). To determine genotype calls we normalized reporter (Rn) data from the cycling stage (option *Analyze Real-Time dRn data*) and enabled the autocaller algorithm. Four homozygotes (two pigs and two wild boars) and six heterozygotes (three pigs and three wild boars) per SNP were sequenced in both directions to check for correct sequence. Once the testing setup was completed, all reference and field samples were analyzed for allelic discrimination at the four SNPs.

### Ethics approval and consent to participate

No animal has been sacrificed for the purposes of this study. Wild boar samples came from regular hunting, according to the Italian (national and regional) laws. Pig samples were meat intended for human consumption, collected from retailers or directly from farmers.

## Results

### Bayesian analysis of STR loci

The Evanno’s Δk method suggested that the best partitioning of our reference dataset of genotypes was K = 2, with wild boars and domestic pigs being assigned to two distinct gene pools. However, the assignment of clusters for K = 2 was not geographically and biologically coherent, failing to separate the European wild boars from the Sardinian subspecies (cf.^36,37^). This was probably due to small sample size for *S. s. meridionalis* (N = 27 vs. N = 137), resulting in lack of Hardy-Weinberg equilibrium. In addition, a critical analysis of the posterior probabilities for higher values of K, the values of α and individual assignment patterns (cf.^31^) led us to refute the partition of the data in two clusters, selecting instead K = 4 as the most meaningful structure of our dataset. The STRUCTURE analysis at K = 4 split the Sardinian wild boars from their continental counterparts and from pigs, and showed better assignment of genotypes in their respective clusters. Based on the cut-off of 0.10, all individuals in the pig sample, except two, showed q-values ranging from 0.900 to 0.994, and were grouped in cluster II with Q value = 0.969 (Table 2). European wild boars were split into clusters I and IV, reaching a cumulative Q = 0.954. Sardinian wild boars were assigned to cluster III with Q = 0.912, and shared ancestry with the continental wild boar clusters I and IV with Q < 0.100. Two reference domestic pigs had q_i_ = 0.244 and 0.192 jointly in wild boar clusters I, III and IV, showing mixed ancestry. We cannot exclude that the non-commercial origin of these samples (they came from field-reared domestic pigs) has yielded this result, that is, we cannot exclude that recent crossbreeding with wild boars has actually occurred. Four reference wild boars showed proportion of membership in their parental population lower than 0.900 and shared ancestry in cluster II (q_i_ = 0.222, 0.236, 0.287 and 0.283, respectively), thus revealing admixture with domestic pigs. Consistent results were obtained from five runs.

**Table 2:** Average probability of membership for reference domestic pig (DP, N = 83), European wild boar (E-WB, N = 137) and Sardinian wild boar (S-WB, N = 27) in the four clusters inferred by STRUCTURE under the admixed model, without prior population information, with correlated allele frequencies.

	Cluster I	Cluster II	Cluster III	Cluster IV
DP	0.010 (0.002–0.075)	0.969 (0.900–0.994)	0.013 (0.002–0.054)	0.008 (0.002–0.017)
E-WB	0.398 (0.004–0.989)	0.016 (0.002–0.096)	0.030 (0.002–0.525)	0.556 (0.005–0.991)
S-WB	0.051 (0.003–0.794)	0.006 (0.002–0.029)	0.912 (0.190–0.993)	0.031 (0.002–0.216)

Ranges for probability of membership are in parentheses.

In a second step, we ran STRUCTURE under the same settings used for the reference samples, including 6 hybrids with known genealogy (Hy_1 to Hy_6) and 15 morphologically putative hybrids (Phe_1 to Phe_15). These genotypes and the six admixed genotypes (two domestic pigs and four wild boars) found *a posteriori* in the reference dataset by previous runs, were excluded from the update of allele frequencies. All hybrids with known genealogy were confirmed, showing proportion of membership in the pig cluster < 0.900 (Table 3). As expected, pig backcross Hy_4 had high q_i_-value (0.830) in the pig cluster, while wild boar backcross Hy_5 had low q_i_-value (0.175) in the pig cluster. Of the F1s, only two had q-values close to the theoretical q = 0.5 (Hy-1 q_i_ = 0.571; Hy-2 q_i_ = 0.592), while Hy_3 and Hy_6 had q_i_-values ranging from 0.299 to 0.766. Four of the morphologically putative hybrids (Phe-1, -4, -9, -11) showed q_i_-values > 0.900 in the wild boar clusters, and were not confirmed as hybrids by the Bayesian analysis of STRs. The remaining morphologically putative hybrids were confirmed, showing q_i_-values < 0.900 (0.120 < q_i_ > 0.888) in their respective parental clusters.

**Table 3:** Results from STRs and SNPs for 6 hybrids with known genealogy and 15 putative hybrids (phenotypically deviating from parental types).

		q_DP_	MC1R_1	MC1R_2	MC1R_3	MC1R genotypes	NR6A1 genotypes
**Hy**_**1**	F1	0.571	A	G	G	E^D2^/E^D2^ E^D2^/E^P1/2/3^	TT
**Hy**_**2**	F1	0.592	G/A	G/A	G	e/E^D2^ e/E^P1/2/3^	TT
**Hy**_**3**	F1	0.766	G/A	G/A	G	e/E^D2^ e/E^P1/2/3^	TT
**Hy**_**4**	F1 × DP	0.830	G/A	G	G	**E**^**+/**^E^D2^ **E**^**+**^/E^P1/2/3^	T/**C**
**Hy**_**5**	F1 × WB	0.175	G/A	G	G	**E**^**+/**^E^D2^ **E**^**+**^/E^P1/2/3^	TT
**Hy**_**6**	F1	0.299	G/A	G	G	**E**^**+/**^E^D2^ **E**^**+**^/E^P1/2/3^	T/**C**
**Phe**_**1**	DP	0.030	A	G	G	E^D2^/E^D2^ E^D2^/E^P1/2/3^	**CC**
**Phe**_**2**	DP	0.540	G/A	G	G	**E**^**+/**^E^D2^ **E**^**+**^/E^P1/2/3^	TT
**Phe**_**3**	WB	0.491	G/A	G	G	**E**^**+/**^E^D2^ **E**^**+**^/E^P1/2/3^	TT
**Phe**_**4**	WB	0.074	G/A	G	G	**E**^**+/**^E^D2^ **E**^**+**^/E^P1/2/3^	TT
**Phe**_**5**	WB	0.213	G	G	G	**E**^**+/**^**E**^**+**^	**CC**
**Phe**_**6**	DP	0.745	A	G	G	E^D2^/E^D2^ E^P1/2/3^/E^P1/2/3^ E^D2^/E^P1/2/3^	T/**C**
**Phe**_**7**	DP	0.392	G	G	G	**E**^**+/**^**E**^**+**^	**CC**
**Phe**_**8**	DP	0.386	G	G	G	**E**^**+/**^**E**^**+**^	**CC**
**Phe**_**9**	WB	0.039	G/A	G	G	**E**^**+/**^E^D2^ **E**^**+**^/E^P1/2/3^	T/**C**
**Phe**_**10**	WB	0.120	G	G/A	G	**E**^**+**^/e	T/**C**
**Phe**_**11**	WB	0.046	G	G	G	**E**^**+/**^**E**^**+**^	**CC**
**Phe**_**12**	WB	0.408	G	G	G	**E**^**+/**^**E**^**+**^	**CC**
**Phe**_**13**	DP	0.315	G/A	G	G	**E**^**+/**^E^D2^ **E**^**+**^/E^P1/2/3^	T/**C**
**Phe**_**14**	WB	0.772	G	G	G	**E**^**+/**^**E**^**+**^	T/**C**
**Phe**_**15**	DP	0.888	G/A	G/A	G	e/E^D2^ e/E^P1/2/3^	T/**C**

DP(WB) = domestic pig (wild boar) putative source population of hybrids; F1 = first generation hybrid; q_DP_ = proportion of membership in the domestic pig cluster obtained by STRUCTURE. Wild-type alleles at *MC1R* and *NR6A1* are in bold.

### SNP assays

Three sites in the *MC1R* gene and one site in the *NR6A1* gene were considered for diagnostic polymorphisms and analyzed through real time PCR assays to discriminate domestic pig, wild boar and their hybrids. We used the Primer Express software with minor changes of the default settings to design *in silico* sets of primers/TaqMan probe that reveal single base compositions. Four sets with the lowest penalty number were ultimately selected to discriminate the alleles. Details on primer and probe sequences, amplicon sizes, nucleotide bases and corresponding alleles are summarized in Table 1. Ten pigs and ten wild boars were first tested for successful amplification of the assays and to establish the Ct cut-off values. Following the default settings, samples with Ct values exceeding 35 were considered negative for all four primers/probe sets. The use of normalized Rn data for genotype calls allowed correct allocation of homozygous and heterozygous genotypes in the allelic discrimination plots. Bidirectional sequencing of alleles from four homozygotes and six heterozygotes matched the expectations and confirmed that the sequences were correct. The whole set of reference samples was eventually analyzed at the four SNPs.

We obtained consensus genotypes at the *MC1R* gene for both pig and wild boar reference samples. A consensus genotype is defined as the allelic composition that corresponds to a given nucleotide base at each single SNP position, and which is concurrently shared by all three SNP positions. For example, the consensus genotype E^+^/E^+^ carried by the 138 wild boars shown in Table 4 is one of the genotypes consistent with the bases G, G, and G at positions MC1R-1, MC1R-2 and MC1R-3, respectively, and is the only genotype that is shared by all three positions. Accordingly, 38 out of 83 pigs beared consensus genotypes E^D2^/E^D2^ and E^D2^/E^P1/2/3^ at *MC1R*, while 45 pigs beared *e*/E^D2^ and *e*/E^P1/2/3^ (Table 4). No wild alleles were identified at *MC1R* in the domestic pig dataset, nor was the allele E^D1^ (corresponding to A at MC1R_3) found in either pigs or wild boars (see also below), suggesting that introgression of alleles from Asian pigs into the Italian domestic and wild gene pools is absent or negligible; (^14,16,28^ but see also^34,38,39^). Of the 164 reference wild boars, 138 showed the wild genotype E^+^/E^+^, and 25 turned out to be hybrids, showing one domestic allele in their genotypes (E^+^/E^D2^ and E^+^/E^P1/2/3^). One wild boar carried a domestic genotype E^D2^/E^D2^ or E^D2^/E^P1/2/3^. The European domestic allele *e*, as well as the Asian domestic allele E^D1^ were absent from hybrid genotypes.

**Table 4:** Genotypes at three positions of *MC1R* gene in the domestic pig (DP) and wild boar (WB) reference dataset. Wild-type alleles are in bold.

	N	MC1R_1		MC1R_2	genotype	MC1R_3	genotype	MC1R consensus genotype(s)
		base	genotype	base		base		
**DP**	83							
	38	A	E^D2^/E^D2^ E^P1/2/3^/E^P1/2/3^ E^D2^/E^P1/2/3^	G	**E**^**+/**^**E**^**+**^ **E**^**+**^/E^D1^ **E**^**+**^/E^D2^ **E**^**+**^/E^P1/2/3^ E^D1^/E^D1^ E^D1/^E^D2^ E^D1^/E^P1/2/3^ E^D2^/E^D2^ E^D2^/E^P1/2/3^	G	**E**^**+/**^**E**^**+**^ **E**^**+**^/e **E**^**+**^/E^D2^ **E**^**+**^/E^P1/2/3^ e/e e^/^E^D2^ e/E^P1/2/3^ E^D2^/E^D2^ E^D2^/E^P1/2/3^ E^P1/2/3^/E^P1/2/3^	E^D2^/E^D2^ E^D2^/E^P1/2/3^
	45	G/A	**E**^**+/**^E^D2^ **E**^**+**^/E^P1/2/3^ E^D1^/E^D2^ E^D1^/E^P1/2/3^ e/E^D2^ e/E^P1/2/3^	G/A	e/**E**^**+**^ e/E^D2^ e/E^D1^ e/E^P1/2/3^	G	**E**^**+/**^**E**^**+**^ **E**^**+**^/e **E**^**+**^/E^D2^ **E**^**+**^/E^P1/2/3^ e/e e/E^D2^ e/E^P1/2/3^ E^D2^/E^D2^ E^D2^/E^P1/2/3^ E^P1/2/3^/E^P1/2/3^	e/E^D2^ e/E^P1/2/3^
**WB**	164*							
	138	G	**E**^**+/**^**E**^**+**^ **E**^**+**^/E^D1^ **E**^**+**^/e E^D1^/E^D1^ e/e e/E^D1^	G	**E**^**+/**^**E**^**+**^ **E**^**+**^/E^D1^ **E**^**+**^/E^D2^ **E**^**+**^/E^P1/2/3^ E^D1^/E^D1^ E^D1/^E^D2^ E^D1^/E^P1/2/3^ E^D2^/E^D2^ E^D2^/E^P1/2/3^	G	**E**^**+/**^**E**^**+**^ **E**^**+**^/e **E**^**+**^/E^D2^ **E**^**+**^/E^P1/2/3^ e/e e^/^E^D2^ e/E^P1/2/3^ E^D2^/E^D2^ E^D2^/E^P1/2/3^ E^P1/2/3^/E^P1/2/3^	**E**^**+/**^**E**^**+**^
	25	G/A	**E**^**+/**^E^D2^ **E**^**+**^/E^P1/2/3^ E^D1^/E^D2^ E^D1^/E^P1/2/3^ e/E^D2^ e/E^P1/2/3^	G	**E**^**+/**^**E**^**+**^ **E**^**+**^/E^D1^ **E**^**+**^/E^D2^ **E**^**+**^/E^P1/2/3^ E^D1^/E^D1^ E^D1/^E^D2^ E^D1^/E^P1/2/3^ E^D2^/E^D2^ E^D2^/E^P1/2/3^	G	**E**^**+/**^**E**^**+**^ **E**^**+**^/e **E**^**+**^/E^D2^ **E**^**+**^/E^P1/2/3^ e/e e/E^D2^ e/E^P1/2/3^ E^D2^/E^D2^ E^D2^/E^P1/2/3^ E^P1/2/3^/E^P1/2/3^	**E**^**+/**^E^D2^ **E**^**+**^/E^P1/2/3^
	1	A	E^D2^/E^D2^ E^P1/2/3^/E^P1/2/3^ E^D2^/E^P1/2/3^	G	**E**^**+/**^**E**^**+**^ **E**^**+**^/E^D1^ **E**^**+**^/E^D2^ **E**^**+**^/E^P1/2/3^ E^D1^/E^D1^ E^D1^/E^D2^ E^D1^/E^P1/2/3^ E^D2^/E^D2^ E^D2^/E^P1/2/3^	G	**E**^**+/**^**E**^**+**^ **E**^**+**^/e **E**^**+**^/E^D2^ **E**^**+**^/E^P1/2/3^ e/e e/E^D2^ e/E^P1/2/3^ E^D2^/E^D2^ E^D2^/E^P1/2/3^ E^P1/2/3^/E^P1/2/3^	E^D2^/E^D2^ E^D2^/E^P1/2/3^

*European and Sardinian wild boars are considered jointly here. See text for details.

All reference pigs showed the domestic allele (T) at the *NR6A1* gene (Table 5), except two individuals from family-owned farms that were pig × wild boar hybrids (T/C). Of these, one had already been identified by STRs. Of 164 reference wild boars, 15 turned out to be hybrids, and one carried a domestic genotype.

**Table 5:** Genotypes at *NR6A1* gene in the domestic pig (DP) and wild boar (WB) reference dataset.

	N	NR6A1 genotype
**DP**	**83**	
	81	TT
	2	T/**C**
**WB**	**164**	
	148	**CC**
	15	T/**C**
	1	TT

The wild-type allele is in bold.

To sum up, the application of four SNP-based tests to our reference dataset (83 pigs and 164 wild boars) revealed that 2 pigs beared wild alleles, 38 wild boars were hybrids and 2 wild boars beared domestic homozygous genotypes. It should be noted that the total number of hybrids mentioned above is not the sum of the hybrids based on *MC1R* and *NR6A1* separately, since some individuals were hybrids at both genes and therefore were calculated only once.

Three out of 6 hybrids with known genealogy were confirmed by SNP polymorphisms at one (*MC1R*, Hy-5) or both genes (Hy-4, Hy-6) (Table 3). Conversely, Hy-1, -2 and -3 showed all domestic genotypes. Of 15 morphologically putative hybrids, 9 were identified as genetically hybrids with one or both genes (Phe-2, -3, -4, -6 and Phe-9, -10, -13, -14, -15, respectively), while 5 (Phe-5, -7, -8, -11, -12) turned out to be pure wild boars. One individual, Phe-1, was identified as domestic pig by *MC1R* polymorphisms and as pure wild boar by *NR6A1*.

### Combined STR and SNP genotyping

When the complete protocol (STRs + SNPs) was applied to the pig reference dataset, the number of hybrids detected increased from 0 and 2 using SNPs and STRs individually (*MC1R* = 0 hybrids identified, *NR6A1* = 2 hybrids, STRs = 2 hybrids), to 3 (Table 6). In the reference wild boar dataset, the number of hybrids (or genetically pure pigs) grew from 4 when using STRs, 16 and 26 when using *NR6A1* and *MC1R*, respectively, to 41 if all markers were employed (few hybrids were identified by more than one system). Hybrids with known genealogy were all genetically identified with the combined protocol (Table 3), while the SNPs-based system failed to reveal hybridization for three of them (Hy-1, -2, -3). Of the putative hybrids, only one (Phe-11) was not confirmed either with STRs or SNPs, and appeared to be a pure wild boar.

**Table 6:** Number of hybrids in the reference dataset based on STRs and SNPs individually, and based on a joined STR + SNP approach.

	N	STRs		SNPs *NR6A1 MC1R*		STRs + SNPs*
		q_DP_ < 0.90	q_WB_ < 0.90	HY_D_	HY_W_	
DP	83	2		2	0	3
WB	164		4	16	26	41

HY_D_ = number of hybrids with domestic alleles; HY_W _= number of hybrids with wild alleles; q_WB_ = proportion of membership in the wild boar clusters; q_DP_ = proportion of membership in the domestic pig cluster. *The total number of hybrids is not the sum of hybrids obtained through STRs and SNPs separately, given that some individuals were identified as hybrids by both systems and therefore were counted only once.

## Discussion

The gene pools of wild and domestic forms of *Sus scrofa* are only partially separated, since domestication has never prevented that a bidirectional natural and human-mediated gene flow occurred between the two^16,34,40^. It may therefore be challenging to identify genetically their hybrids, and sometimes it is also difficult to distinguish the parental taxa. Morphology often does not reveal any clue of hybridization, nor does a wild type phenotype guarantee that we are dealing with a pure wild boar. For example, the wild boar samples analyzed in this study were all provided by hunters, that identified them in the field as true wild boars, clearly without detecting strong deviations from the wild type phenotype.

Domestic pigs and wild boars share genetic polymorphisms at known loci^11,34^, therefore no single diagnostic markers are available that individually are able to distinguish the two subspecies and their hybrids with certainty (cf.^41^). Hence the need for combined approaches of multiple genetic markers which, on the one hand increase diagnostic power, and on the other are technically and financially feasible to laboratories that perform diagnosis and not research. Our genotyping procedure has precisely this twofold purpose: it associates the analysis of STRs, which every molecular-oriented laboratory can currently be equipped to handle, to real-time-PCR-based assays of single SNPs, which are simple, inexpensive, sensitive and extremely fast to perform, so being easily adaptable for routine large scale sample processing.

When applying both genetic systems, the number of hybrids that we found (i.e. positives) in our pig dataset increased from 0% (using *MC1R* individually), 24% (*NR6A1*), 24% (STRs) to 36%. Comparable results were obtained for the reference wild boars, for which the percentage of positives raised from 2.4% (STRs), 10% (*NR6A1*), 16% (*MC1R*) to 25% under the combined approach. Regarding the field samples, the protocol correctly identified all known and putative hybrids, with the exception of one, which proved a wild boar. However, we cannot exclude that its non-wild morphological traits were misinterpreted in the field, and that it actually was a pure wild boar.

STR genotyping detected fewer hybrids than was expected if we compared the results from both *NR6A1* and *MC1R* genes. A plausible explanation is based on the observation that STR panels detect efficiently recent hybridization events, but with greater difficulty or not at all later-generation hybrids, especially when parental gene pools are poorly differentiated, showing many shared ancestral polymorphisms (^16,34^). The proportion of genome not coming from own parental population quickly dilutes, becoming no longer detectable in further-back-in-time hybrids. It is therefore possible that non-recent hybridization between pigs and wild boars cannot be revealed by our 20-STR multilocus system. *A fortiori*, a combined approach of multiple markers with different molecular and evolutionary characteristics is much needed for discrimination purposes.

In addition to demonstrating great efficacy, our protocol is efficient, because it involves real-time qualitative PCR assays for the allelic discrimination of few diagnostic SNPs, thus providing to be an excellent alternative to more hardworking, time-consuming, costly techniques, such as PCR-RFLP-based analysis or sequencing of mitochondrial and nuclear genes^11,22,23,27–29,42^.

The diagnostic procedure presented here has a wide range of forensic applications, including the fields of food adulteration and food safety. Pig meat is sometimes illegally sold as wild boar meat, which is valuable and therefore more expensive. The availability of a reliable molecular protocol to detect domestic alleles in game products is of great help to prosecute commercial frauds of this type. Furthermore, *Sus* meat, if not recognized as game meat, is consequently not adequately monitored for diseases before consumption by humans. This is potentially highly dangerous for public health, as wild boar meat can be source of serious zoonotic diseases, such as trichinellosis, hepatitis E, tubercolosis and leptospirosis^43^. Additionally, this molecular procedure becomes an essential investigative tool in wildlife forensics. Very often wild boars are illegally farmed and kept in captivity, although brazenly declared as pigs. Sometimes farmers deliberately crossbreed their domestic animals with wild boars to improve meat quality, thus committing a crime against local laws on the protection and conservation of wildlife^44,45^. Furthermore, if escaped from captivity, farmed hybrids contribute to increase the risk of genetic introgression in wild populations^34,39^.

Our protocol is currently a good cost/benefit compromise to identify wild and domestic forms of *S. scrofa* and their hybrids. However, in the near future, more powerful methodologies, like high-density genome-wide SNP analysis, for example through the Illumina porcine SNP chip assay^46^, will be more and more affordable, while extremely promising in the identification of private wild and domestic polymorphisms for the routine diagnosis of subspecies and their recent and past hybrids^39^.

## Conclusions

The results of our study showed that a combined approach of multiple molecular markers is effective in genetically discriminating between wild boar and domestic pig, assigning individual genotypes to one or the other parental gene pool, identifying the hybrids between the two forms. Used jointly, multiplex STR-typing and real time PCR-based assays of single polymorphisms in the *NR6A1* and *MC1R* genes outperformed the same marker systems used individually, showing significant reduction in false negative results. Our protocol is an effective diagnostic procedure that can be adopted by most forensic laboratories to face the complex issue of differentiating pig and wild boar.

## Supplementary information

Table S1. Online MC1R sequences used in the alignment to design primers and probes for real time PCR assays.

## Data Availability

All data generated or analyzed during this study are included in this published article.
