# Supplementary material for: Matching STR and SNP genotyping to discriminate between wild boar, domestic pigs and their recent hybrids for forensic purposes
Source: Sci Rep. 2020 Feb 21;10:3188. doi: 10.1038/s41598-020-59644-6 (PMC7035276; doi:10.1038/s41598-020-59644-6)
Supplement: Supplementary file 1 — Table S1. Online MC1R sequences used in the alignment to design primers and probes for real time PCR assays. [file 41598_2020_59644_MOESM1_ESM.docx]

**Supplementary information**

**Matching STR and SNP genotyping to discriminate between wild boar, domestic pigs and their recent hybrids for forensic purposes**

Rita Lorenzini^1^*, Rita Fanelli^1^, Francesco Tancredi^2^, Antonino Siclari^3^, Luisa Garofalo^1^

1 Istituto Zooprofilattico Sperimentale delle Regioni Lazio e Toscana "M. Aleandri", Centro di Referenza Nazionale per la Medicina Forense Veterinaria, Via Tancia 21, 02100 Rieti, Italy. [rita.lorenzini@izslt.it](mailto:rita.lorenzini@izslt.it), [rita.fanelli@izslt.it](mailto:rita.fanelli@izslt.it), [luisa.garofalo@izslt.it](mailto:luisa.garofalo@izslt.it)

2 Istituto Zooprofilattico Sperimentale delle Regioni Lazio e Toscana "M. Aleandri", Via Tancia 21, 02100 Rieti, Italy. [francesco.tancredi@izslt.it](mailto:francesco.tancredi@izslt.it)

3 Ente Parco Nazionale dell'Aspromonte

Via Aurora 1, 89057 Gambarie di S. Stefano in Aspromonte, Reggio Calabria, Italy.

[antonino.siclari@parcoaspromonte.gov.it](mailto:antonino.siclari@parcoaspromonte.gov.it)

* Correspondence: Istituto Zooprofilattico Sperimentale delle Regioni Lazio e Toscana "M. Aleandri", Centro di Referenza Nazionale per la Medicina Forense Veterinaria, Via Tancia 21, 02100 Rieti, Italy. Tel.: ++39 0746 201599; Fax: ++39 0746 201642

E-mail: [rita.lorenzini@izslt.it](mailto:rita.lorenzini@izslt.it) (Rita Lorenzini).

**Table S1.** Online MC1R sequences used in the alignment to design primers and probes for real time PCR assays.

| **Acc. number** | **Reference** | **MC1R allele** |
| --- | --- | --- |
| AF082487 | Kijas et al. 1998 | E^D1^ |
| AF082488 | Kijas et al. 1998 | E^+^ |
| AF082490 | Kijas et al. 1998 | E^+^ |
| AF082489 | Kijas et al. 1998 | E^D1^ |
| EU443672 | Fang et al. 2009 | E^D1^ |
| EU443682 | Fang et al. 2009 | E^D1^ |
| EU443645 | Fang et al. 2009 | E^+^ |
| EU443691 | Fang et al. 2009 | *e* |
| EU443692 | Fang et al. 2009 | *e* |
| EU443722 | Fang et al. 2009 | E^P2^ |
| EU443685 | Fang et al. 2009 | E^D2^ |
| EU443700 | Fang et al. 2009 | E^P2^ |
| EU443726 | Fang et al. 2009 | E^P3^ |
| AM231528 | Du et al. 2006, unpublished | E^D1^ |
| AY365254 | Cho et al. 2004, unpublished | E^+^ |
| AY916523 | Shi et al. 2006, unpublished | *e* |
| AY365250 | Cho et al. 2004, unpublished | *e* |
| AY365251 | Cho et al. 2004, unpublished | E^D2^ |
| AY365253 | Cho et al. 2004, unpublished | E^D2^ |
| AY916524 | Shi et al. 2006, unpublished | *e* |
| DQ191187 | Cho et al. 2006, unpublished | E^+^ |
| DQ191204 | Cho et al. 2006, unpublished | E^+^ |

**References**

Kijas, J. M., *et al.* Melanocortin receptor 1 (*MC1R*) mutations and coat color in pigs. *Genetics*. **150**, 1177-85 (1998).

Fang, M. Y., Larson, G., Ribeiro, H.S., Li, N. & Andersson, L. Contrasting mode of evolution at a coat color locus in wild and domestic pigs. *PLoS Genetics*. **5**, 1-6 (2009).
